# Editorial: Ensuring public health: the active role of healthcare professionals

**DOI:** 10.3389/fpubh.2026.1822156

**Published:** 2026-03-27

**Authors:** Martina Giusti, Helena José, Ana M. Pires, Esperanza Begoña García-Navarro, Guglielmo Bonaccorsi, Niccolo Persiani

**Affiliations:** 1 Department of Experimental and Clinical Medicine, University of Florence, Florence, Italy; 2 Centro Studi SAPIS Foundation, Italian National Federation of Orders of Radiographers and Technical, Rehabilitation, and Prevention Health Professions Research Centre, Rome, Italy; 3 RISE – Health Research Network, Porto, Portugal; 4 Nursing Research Innovation and Development Centre of Lisbon (CIDNUR), Nursing School of Lisbon (ESEL), Lisbon, Portugal; 5 Escola Superior de Saùde Atlântica, Barcarena, Portugal; 6 Atlântica, Instituto Universitário, Barcarena, Portugal; 7 Nursing Department, University of Huelva, Huelva, Spain; 8 Department of Health Sciences, University of Florence, Florence, Italy

**Keywords:** Health management, innovation, integration, multidisciplinary teams, multiprofessional approach, research-intervention, social and healthcare services, task-shifting and task-sharing

The Research Topic “*Ensuring public health: the active role of healthcare professionals*,” hosted by *Frontiers in Public Health*, reached a wide audience by providing a platform not only for clinical research at micro level, but also for studies exploring the integration of social and healthcare services at the meso and macro levels of health systems. It actively encouraged contributions from healthcare professionals (HCPs), especially belonging to the allied health disciplines. For this reason, the Research Topic sought to promote greater integration between the Public Health Education and Promotion and Health Economics subsections of *Frontiers in Public Health*.

When this Research Topic was first proposed more than two years ago, the Editors aimed to gather evidence on how research conducted by HCPs—focused on the continuous optimization of services delivery processes, health management, and the implementation of innovation through multiprofessionals multidisciplinary approaches-, could strengthen integrated social and healthcare services within personalized care pathways. There was a clear need to synthesize experiences aimed at fostering proactive and coordinated care for every citizen, encouraging health and social care systems to assume responsibility not only for addressing illness, but also for ensuring equitable protection of health and promoting a higher quality of life. This vision extends beyond merely meeting healthcare needs, embracing instead a broader commitment to prevention, integration, and long-term wellbeing for all.

The proposed topic generated considerable interest within the scientific community. As of February 28th, 2026, the Research Topic had recorded 78,259 total views, 17,443 article downloads and 4,860 topic views, reflecting engagement from researchers worldwide: America (39 %), Asia (44 %), Europe (16 %), Africa (1 %), and Oceania (0,4%).The interest was further demonstrated by the submission of 64 manuscripts, of which 36 were ultimately accepted for publication: 1 Brief Research Report, 1 Community Case Study, 1 Data Report, 24 Original Research articles, 2 Perspectives, 1 Policy Brief, 2 Reviews, 3 Study Protocols, and 1 Systematic Review. Overall, 223 authors contributed to these publications. The majority were affiliated with institutions in Italy (30.5%) and China (24.7%), followed by the United Kingdom (6.3%) and Brazil (5.4%), while authors from other countries each represented less than 2% of the total.

Published articles can be grouped into five thematic categories (Table 1):

- Organizational contributions (1). The articles in this category present studies aimed at supporting the reorganization and optimization of social and healthcare service delivery models, with the objective of enhancing efficiency and effectiveness.
- Evidence based practice—EBP (2). This category includes studies that demonstrate the impact of applying Evidence-Based Medicine principles in professional practice, particularly in the development and implementation of protocols and procedures designed to ensure safety and quality in the provision of social and healthcare services.
- Educational initiatives (2). The research grouped here reports evidence derived from educational projects conducted by HCPs and targeted at service users, with the goal of improving health knowledge and promoting informed engagement.

a. Communication (3). Studies in this category focus on interventions aimed at strengthening health communication as a strategic tool for health promotion and for safeguarding equitable access to social and healthcare services.

- Professional aspects (4). This category encompasses studies that explore defining characteristics of specific professional profiles, examining their competencies, scope of practice, and potential evolution in response to changes within healthcare systems.
- Training (5). The articles included in this category present studies that evaluate the impact of training programs designed for HCPs, with particular attention to their effectiveness in enhancing competencies, supporting professional development, and promoting alignment with evidence-based standards of practice.

**Table 1:** Categories of Research Topic “Ensuring public health: the active role of healthcare professionals.”

Categories	Article type	Title
Organizational contributions	Review	*Implementation of cross-sectoral rehabilitation in the Nordic countries: a scoping review* (Bøgard et al.)
	Perspective	*Leveraging new methodologies for public health crisis management* (Khalil et al.)
	Community case study	*IdentIRCCS. The description of healthcare professionals employed within IRCCS University Hospital of Bologna* (Giusti, Quirini et al.)
	Original research	*Enhancing the feeding journey of the preterm infant in the NICU: the STAmmi VICINO pathway as a model of integrated and individualized neurodevelopmental care* (Naboni et al.)
	Brief research report	*Effectiveness and cost savings of employing speech therapists in a home artificial nutrition unit* (Battista et al.)
	Original research	*The emergency medical services network's response to the COVID-19 pandemic in Albania* (Persiani et al.)
	Original research	*Facilitators and barriers to chronic non-communicable disease management under family doctor contracting services in China* (Jiang et al.)
	Original research	*Economic impact of patients with medical evacuation in remote islands: a case study in Matsu Islands* (Hseih et al.)
	Original research	*Analysis and assessment of biomedical scientists' needs for clinical laboratory: activity-based management as an evaluation methodology* (Bizzoni et al.)
	Original research	*Optimization of nursing staff standards in the perioperative settings of the IRCCS University Hospital of Bologna. An improvement project* (Cenacchi et al.)
Evidence based practice	Review	*Malignant fungating wounds assessment in palliative care: a scoping review* (Nigrelli et al.)
	Study protocol	*Study protocol for identification of patients with risk of cognitive impairment in advanced pharmaceutical care in a community pharmacy* (Macekova et al.)
	Study protocol	*Virtual reality cognitive remediation tool for individuals with mild cognitive impairment: study protocol for a feasibility randomized clinical trial* (Perra et al.)
	Policy brief	*A multi-level approach to reduce exploding type 2 diabetes in Pakistan* (Jamil et al.)
	Original research	*A cross-sectional study of evidence-based practice and association factors among nurses in public health facilities of Dessie city, Northeast Ethiopia* (Yimam et al.)
	Original research	*How exercise frequency affects BMI: a nationwide cross-sectional study exploring key influencing factors, including dietary behaviour* (Zhang et al.)
	Original research	*Urban household food insecurity and pediatric malnutrition: evidence from the Divina Providencia Hospital in Luanda, Angola* (Carbone et al.)
Educative initiatives	Systematic review	*Interventions on informal healthcare providers to improve the delivery of healthcare services in low-and middle-income countries: a systematic review* (Das et al.)
	Data report	*Interculturality, public health and health education: data report based on the Virtual Learning Environment of the Brazilian Health System (AVASUS)* (Sanara da Cunha et al.)
	Original research	*The role of nutrition education in the pharmacy curriculum using the example of knowledge about the health benefits of nuts* (Stević et al.)
	Original research	*Development of a self-care scale for women with polycystic ovary syndrome: a methodological approach* (Kim)
	Original research	*Health literacy assessment and analysis of influencing factors in pregnant women with gestational diabetes mellitus in Southwest China* (Tang et al.)
	Original research	“*NE@R”: a new resource to promote preterm infants' development through parents-delivered guided play* (Trussardi et al.)
	Study protocols	*The needs of family caregivers providing palliative home care in Portugal: a multi-stage mixed methods study protocol* (Laranjeira et al.)
Communication	Perspective	*Promoting health equity through cross-sector strategies: the integration of communication public health* (Lopez et al.)
	Original research	*Experiences of health information-seeking among patients with cancer in China: a qualitative study* (Wu et al.)
	Original research	*Assessing the scientific quality of radiotherapy popularization videos on BiliBili and TikTok: a cross-sectional study* (Han et al.)
Professional aspects	Original research	*A comprehensive cross-sectional study on the current status of proactive health awareness among Guangxi healthcare workers and its influencing factors: understanding from both individual and variable viewpoints* (Chen and Guan)
	Original research	*Implementing resilience-based interventions for healthcare employee well-being: evidence from the pandemic crisis* (Kosykarz et al.)
	Original research	*Italian healthcare professionals' role in advancing reforms within the Italian National Healthcare System, as outlined in the National Recovery and Resilience Plan* (Giusti, Beux et al.)
	Original research	*Associations between burnout and career disengagement factors among general practitioners: a path analysis* (Grigoroglou et al.)
	Original research	*Resilience and professional identity among young healthcare workers in a Shanghai megahospital during COVID-19: a cross-sectional study* (Xu et al.)
Training	Original research	*Psychometric evaluation of the Chinese version of self-assessment scale for the community- based and emergency practice among medical students* (Hu, Zheng et al.)
	Original research	*Moral judgment competence of midwifery students and its influencing factors: a cross-sectional study* (Hu, Fan et al.)
	Original research	*Professionalization trajectory of healthcare interpreter certification in the context of the language industry: a comparative study of industry-led and government-led models, with implications for China* (Hu and Jiang)
	Original research	*Evaluation and analysis of theoretical knowledge proficiency and practical skills among parasitic disease practitioners in Hainan province: a cross-sectional survey* (Liang et al.)

It is interesting to observe that most of the identified categories (four out of six) are not attributable to a single professional profile but rather are cross-functional across different healthcare professions. This highlights the inherently multidisciplinary and collaborative nature of the contributions included in this Research Topic.

The relevance and impact of the articles included in this Research Topic are reflected in the steadily increasing number of citations they continue to receive, confirming the scientific and practical significance of the topic addressed.

An in-depth analysis of the article within the “Organizational contributions” category demonstrates the strong commitment of HCPs to organizational research. These studies show that HCPs act as promoters of improvement and optimization initiatives aimed at enhancing efficiency and effectiveness. In doing so, they contribute directly to strengthening the quality and safety of care, as well as to reinforcing their strategic role within the governance of social and health services. Drawing from their frontline experience, these professionals propose solutions that go beyond purely technical interventions designed to address operational issues within a single unit or hospital. Instead, they promote improvement and optimization processes at all levels of healthcare governance. Their contributions operate bottom-up, influencing organizational development at local, national, and even international levels. Moreover, research in the health economics and management domain provides HCPs with opportunities to engage in international comparisons regarding the performance of leadership and managerial functions (6). These roles are increasingly defined within globally shared frameworks, helping to overcome challenges related to the heterogeneity in how professional roles are expressed and regulated across different countries. By situating their work within an international context, HCPs contribute to a more harmonized and strategically aligned understanding of their evolving responsibilities in health systems worldwide.

A substantial number of submissions focus on Evidence-Based Practice (EBP) to the extent that a dedicated category was created to accommodate them. These studies aim to share, within the international scientific community, the diverse approaches through which EBP principles are implemented in real-world healthcare settings. While Evidence-Based Medicine (EBM) can be more readily applied to specific clinical conditions or clearly defined interventions—given its grounding in validated research evidence—the operationalization of broader EBP principles is far more complex. Translating EBM into standardized protocols and procedures that can be consistently implemented across different countries is particularly challenging. This difficulty arises not only from the varying degrees of professional autonomy granted to healthcare practitioners in different national contexts, but also from the heterogeneity in the structure, governance, and management of health systems at all levels. Therefore, there is no universally unambiguous definition of many healthcare professional profiles (7), particularly within the allied health professions (3). Although there is a generally shared international understanding of the roles and responsibilities of professions such as nurses or midwives, this clarity does not extend to many other professionals working in rehabilitation, diagnostics, and prevention. Even within the European context alone, the number and classification of recognized health professions differ significantly from country to country. Furthermore, when the same professional title exists across multiple countries, the scope of practice and associated responsibilities often vary—sometimes substantially. In this context, it becomes essential for HCPs to demonstrate how the protocols and procedures they implement within their local settings align with internationally recognized guidelines. Providing such evidence not only strengthens the scientific robustness of their practice but also contributes to greater transparency, comparability, and harmonization across health systems (5).

Awareness of the specific role of each health profession in the context of intervention and, more generally, of the contribution he can make to the international scientific community to which it belongs, is the basis of all the papers collected in the “Professional aspects” category. It is precisely the professional skills that give uniqueness and dignity to each profession, making it indispensable in a multi-professional and multidisciplinary work team. Only by assuming this awareness by most representatives of a profession it is possible to assess the state of the art of the profession itself, or how it can keep up with the frenetic evolution that characterizes the health sector. By identifying critical issues and strengths, as well as opportunities and threats, it is possible to develop solutions that make professions progress over time, ensuring their ever-greater affirmation and growth over time (4, 8).

Continuous training is certainly the simplest intervention that allows HCPs to keep up with the times, i.e., operating in line with EBM and EBP. For this to happen, however, it is necessary to verify the effectiveness of these interventions over time and, where the results are not fully satisfactory, to introduce and validate the use of educational innovations (for example psychometric evaluation, moral judgments or alternative communicative approaches) to guarantee the pursuit of the set objective. Manuscript included in the “Training” category give evidence of the continuous attention to training not only of students in the health professions but also of professionals (3).

For example, starting from joint training in areas of common interest, the collaborative spirit of individual professionals in multi-professional and multidisciplinary working groups can be strengthened and the organizational and management models of the various operational settings can be oriented toward promoting it. In this regard, an area of interest common to all health professions, which bypasses the differences in recognition of professions in individual national contexts and at the same time enhances the specific contribution of each professional, is the development of information and educational campaigns aimed at the general population or specific targets. The category “Educative initiatives” (2) and the sub-category “Communication” (9) collected manuscript properly referred to the promotion of information and educational campaigns by HCPs. In fact, in daily activity, each professional must be able to effectively inform and, in specific cases, to educate users on the optimal use of the social and health service they provide. In health systems all over the world, this expertise is systematized by directly entrusting health professionals with the implementation of information and education campaigns so that users take an active role in the processes of integrated care in line with the recommendations of the WHO (10). These interventions serve a dual purpose: they not only help users navigate the various services more effectively and use them appropriately but also contribute to making services more “user-friendly” by fostering the development of health-literate healthcare organizations (1). At a time when social and health systems are confronted with major global challenges—such as climate change, geopolitical tensions, population aging, and rapid digital innovation—individuals increasingly risk feeling overwhelmed and disoriented. In designing public information and education campaigns aimed not only at protecting health but also at promoting overall wellbeing, it is therefore essential to consider people's emotional states and psychological resilience.

In this context, HCPs —who are daily engaged in supporting the psycho-physical balance of the individuals they assist—are uniquely positioned to contribute, within multiprofessional teams, to the development of information and educational initiatives that are effective not only from a technical and scientific perspective, but also from a relational and psychosocial one (11).

Through this Research Topic, the Editors believe they have achieved their primary objective: to highlight and strengthen the recognition of the active role played by health professionals—particularly those belonging to the allied health disciplines—in healthcare management, research-intervention processes, and the introduction of innovation within health systems. In doing so, the Issue has brought to the forefront the commitment of allied health professionals as key drivers of cultural change in integrated social and healthcare sectors, fostering transformation both within and beyond healthcare organizations. The interventions and experiences documented in these contributions demonstrate how proactive, integrated, and increasingly standardized models of care can be implemented, with the primary aim of protecting and promoting individual health, and only subsequently, when necessary, providing treatment. This perspective reflects a shift from a reactive model of care to one centered on prevention, health promotion, and integrated responsibility.

Every healthcare professional is therefore called upon to contribute at all organizational levels of the health system and throughout every phase of the care pathway. This requires synergistic collaboration with colleagues and the implementation of mature and well-structured task-shifting processes, grounded in mutual recognition of competencies and shared accountability. However, the full realization of this vision remains partially hindered by the persistent lack of clarity and uniformity in the definition of professional profiles across different contexts. Variability in professional responsibilities and degrees of autonomy continues to limit the possibility of fully standardizing practice at the international level. Addressing this structural challenge represents a crucial step toward consolidating the strategic role of all health professions in the ongoing evolution of health systems.
